# Urinary calcium-to-citrate ratio predicts kidney stone risk in children under the age of two years

**DOI:** 10.1007/s00467-026-07191-z

**Published:** 2026-02-11

**Authors:** Utku Dönger, Meraj Alam Siddiqui, Aysun Çaltık Yılmaz, Caner İncekaş, Esra Baskın

**Affiliations:** 1 Department of Pediatrics, Faculty of Medicine, Başkent University, Ankara, Turkey; 2 Department of Pediatric Nephrology, Etlik City Hospital, Ankara, Turkey; 3 Department of Biostatistics, Başkent University, Ankara, Turkey; 4 Department of Pediatric Nephrology, Faculty of Medicine, Başkent University, Ankara, Turkey

**Keywords:** Calcium-to-citrate ratio, Hypercalciuria, Hypocitraturia, Pediatric urolithiasis, Urinary stones, Infants

## Abstract

**Background:**

The urinary calcium-to-citrate (Ca/Cit) ratio has emerged as a useful indicator of lithogenic risk in older children; however, no reference data exist for infants and toddlers. This study aimed to evaluate whether the spot Ca/Cit ratio can distinguish stone-forming from non–stone-forming children under 24 months of age and to assess its diagnostic performance compared with conventional urinary markers.

**Methods:**

This retrospective single-center study included 181 children aged 1–24 months who underwent metabolic evaluation and ultrasonography at their first presentation to a tertiary pediatric nephrology clinic between 2012 and 2024. Based on urinary calcium excretion and ultrasonographic findings, participants were categorized as normocalciuric stone-free controls (*n* = 57), hypercalciuric stone-formers (*n* = 29), or non-hypercalciuric stone-formers (*n* = 95). Spot urine calcium, citrate, and related biochemical ratios were analyzed. The diagnostic accuracy of the Ca/Cit ratio for predicting stones was assessed using receiver operating characteristic (ROC) analysis.

**Results:**

The Ca/Cit ratio differed significantly across groups, with the highest levels observed in hypercalciuric stone-formers (0.46 mg/mg) compared with controls (0.17 mg/mg; *p* < 0.001) and non-hypercalciuric stone-formers (0.31 mg/mg; *p* < 0.001). A Ca/Cit threshold > 0.23 mg/mg (≈ 1.10 mmol/mmol) demonstrated moderate diagnostic ability for stone detection (AUC 0.695; 95% CI 0.613–0.785), yielding 66.1% sensitivity and 63.2% specificity. Age showed no meaningful correlation with Ca/Cit values. Normocalciuric stone-free children provided an age-appropriate reference distribution for Ca/Cit ratios.

**Conclusions:**

In infants and toddlers evaluated for suspected urinary stone disease, the Ca/Cit ratio offers moderate discriminatory power and may serve as a practical adjunctive marker of stone risk. A ratio > 0.23 mg/mg (≈ 1.10 mmol/mmol) appears to indicate increased lithogenic potential. Larger prospective studies are needed to validate reference intervals and refine clinically applicable cut-off values for this young age group.

**Graphical Abstract:**

**Figure:**
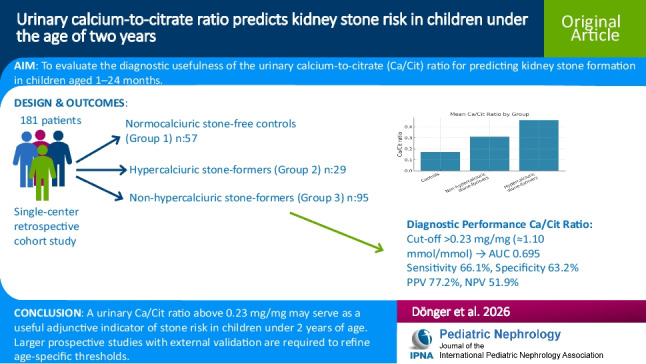
A higher resolution version of the Graphical abstract is available as Supplementary information

**Supplementary Information:**

The online version contains supplementary material available at 10.1007/s00467-026-07191-z.

## Introduction

Recent data suggest that the prevalence of childhood urolithiasis ranges between 5 and 10% [1, 2]. The pathogenesis of stone formation is closely associated with decreased urinary concentrations of natural inhibitors such as citrate, magnesium, and pyrophosphate [3]. Citrate, in particular, plays a critical role by inhibiting spontaneous nucleation and crystal growth of calcium oxalate and calcium phosphate, as well as the heterogeneous nucleation of calcium oxalate on monosodium urate crystals [4]. It has been estimated that citrate accounts for approximately 50% of the inhibitory activity against calcium phosphate crystallization in urine [5].

Among emerging tools for assessing lithogenic risk, the urinary calcium-to-citrate (Ca/Cit) ratio has garnered attention. The evaluation of urinary calcium levels in isolation may not yield an adequately precise estimation of the likelihood of stone formation. The simultaneous assessment of urinary calcium and citrate concentrations has been shown to enhance diagnostic accuracy in patients with suspected calcium stone disease [6, 7]. Although the 8th edition of *Pediatric Nephrology* provides reference values for the Ca/Cit ratio in children older than two years, no such normative data are currently available for children under the age of two [8].

The primary objective of this study was to evaluate whether the Ca/Cit ratio in spot urine can discriminate between stone-forming and non-stone-forming children under 2 years of age, and to compare its diagnostic performance with that of the conventional calcium-to-creatinine (Ca/Cr) ratio. To this end, the Ca/Cit ratio was compared in three groups of children aged 1 month to < 24 months: normocalciuric stone-free controls, hypercalciuric stone-formers, and non-hypercalciuric stone-formers.

## Materials and methods

This single-center retrospective study was conducted at the Pediatric Nephrology outpatient clinic of Başkent University Ankara Hospital between January 2012 and December 2024. It reviewed a cohort of patients aged 1 month to < 24 months of age. Our center serves as a tertiary pediatric nephrology referral clinic in Türkiye, receiving children both from within our institution and from external hospitals with a preliminary diagnosis or suspicion of urinary stone disease. The data pertaining to patients were obtained from the hospital's electronic database. From the medical records, we extracted the following variables: age, sex, body weight, consanguinity, family history of urolithiasis, use of vitamin D prophylaxis, breastfeeding history, use of medications affecting mineral metabolism, use of potassium or sodium citrate, serum BUN, creatinine, sodium, potassium, calcium, phosphorus, magnesium, venous pH and bicarbonate, uric acid, spot urinary Ca/Cr, citrate-to-creatinine (Cit/Cr), oxalate-to-creatinine (Ox/Cr), uric acid-to-creatinine (UA/Cr) ratios, and ultrasonographic findings. These measurements constituted the standard metabolic kidney stone risk evaluation protocol applied to all study participants (both stone-formers and stone-free controls) at their first visit.

At their first presentation to our center, all potentially eligible children underwent kidney and urinary tract ultrasonography and spot urine testing for calcium and citrate as part of the metabolic evaluation. For inclusion in the present study, children were required to be 1 month to < 24 months of age and to have both a same-episode urinary system ultrasonography and spot urinary Ca/Cr and Cit/Cr ratios recorded in the database. Spot urine calcium and citrate levels measured at the initial presentation, together with concurrent urinary system ultrasonography findings, were retrospectively evaluated for a total of 181 pediatric patients.

Based on the presence of hypercalciuria and urinary stones, the patients were categorized into three groups. Group 1 (*n* = 57) consisted of children who were evaluated in the pediatric nephrology outpatient clinic with a preliminary diagnosis of urinary stone disease but were found not to have urinary stones on ultrasonography at their first evaluation in our hospital and had normal urinary calcium excretion (normocalciuric); these children served as the stone-free control group. Group 2 (*n* = 29) included children with both hypercalciuria and urinary stones, while Group 3 (*n* = 95) comprised children with urinary stones but without hypercalciuria. Urinary stones were defined radiologically by the presence of posterior acoustic shadowing, high echogenicity, and size greater than 2 mm [6]. Hypercalciuria was defined as a spot urine Ca/Cr ratio (mg/mg) exceeding the age-specific reference range [8].

Spot urine samples were analyzed for calcium, citrate, oxalate, and uric acid. Urinary Ca/Cr and UA/Cr ratios were expressed as milligrams of solute per milligram of creatinine (mg/mg), whereas Ox/Cr and Cit/Cr ratios were expressed as milligrams of solute per gram of creatinine (mg/g). The Ca/Cit ratio was calculated by dividing urinary calcium (mg/dL) by urinary citrate (mg/dL) [6]. For conversion of Ca/Cit values from mg/mg to mmol/mmol, molecular weights of calcium (40.08 g/mol) and citrate (192.12 g/mol) were used, and values were multiplied by a factor of 4.79.

Patients who had undergone surgical treatment due to urinary tract malformations, those with functional abnormalities of the gastrointestinal system, those using medications affecting mineral metabolism (such as corticosteroids, diuretics, or anticonvulsants), and patients with kidney stones secondary to infection, renal tubular acidosis, or cystinuria were excluded from the study. In addition, children who were already receiving potassium or sodium citrate therapy for any indication at the time of urine sampling were excluded from the study. Children with a documented urinary tract infection (UTI) at the time of urine sampling, as well as those born prematurely who had required neonatal intensive care unit (NICU) admission, were also excluded from the cohort. This retrospective study was approved by the Ethics Committee of Başkent University (Approval No: KA24/402, Date: 19 December 2024). The requirement for informed consent was waived due to the retrospective design of the study.

## Statistical analysis

Descriptive statistics were presented as frequencies and percentages for categorical variables, and as means ± standard deviations (SD) or medians with interquartile ranges (IQR) for continuous variables, based on data distribution. Normality of continuous variables was assessed using both the Kolmogorov–Smirnov and Shapiro–Wilk tests. Since most variables did not follow a normal distribution, non-parametric tests were applied for group comparisons. The Kruskal–Wallis H test was used to compare continuous variables across the three groups. Where significant differences were detected, pairwise comparisons were performed using the Mann–Whitney U test. To adjust for multiple comparisons, a Bonferroni correction was applied, resulting in an adjusted significance threshold of *p* < 0.0033. For categorical variables, group comparisons were made using the Chi-square test or Fisher’s exact test, as appropriate. To evaluate the diagnostic performance of the Ca/Cit ratio for predicting the presence of urinary stones on ultrasonography, receiver operating characteristic (ROC) curve analysis was performed. The area under the ROC curve (AUC) was reported along with 95% confidence intervals (CI). Optimal cut-off values were determined using sensitivity, specificity, positive predictive value (PPV), and negative predictive value (NPV). The relationship between age and the Ca/Cit ratio was examined using Spearman’s rank correlation coefficient and visualized with a scatter plot (Ca/Cit vs. age). All statistical tests were two-tailed, and a p-value of < 0.05 was considered statistically significant unless otherwise adjusted. All statistical analyses were conducted using IBM SPSS Statistics for Windows, Version 25.0 (IBM Corp., Armonk, NY, USA).

## Results

### Baseline characteristics

A total of 181 children between 1 month and < 24 months of age were included in the study. Of these, 79 (43.6%) were female and 102 (56.4%) were male. Hypercalciuria was observed in 29 patients (16.02%), while 152 (83.98%) had urinary calcium levels within normal limits. Hypocitraturia was identified in 30 children (16.57%), whereas 151 (83.43%) had normal urinary citrate levels. Consanguinity was reported in 23 patients (12.71%). A positive family history of urinary stone disease was noted in 97 children (53.59%), and 84 (46.41%) had no such history. The median age at first nephrology evaluation was 6.0 months (IQR 1.0–24.0), and the mean body weight was 7.25 ± 2.62 kg. There were no statistically significant differences between the groups in terms of breastfeeding during the first 6 months of life and the use of vitamin D prophylaxis during the first year of life (*p* > 0.05).

Laboratory parameters revealed a mean blood urea nitrogen level of 8.67 ± 4.94 mg/dL, and a median serum creatinine level of 0.43 mg/dL (IQR: 0.18–0.79). Serum electrolytes were within the normal reference ranges, with mean sodium at 136.86 ± 2.35 mmol/L, potassium at 4.75 ± 0.51 mmol/L, calcium at 10.33 ± 0.92 mg/dL, phosphorus at 5.48 ± 0.70 mg/dL, and magnesium at 2.16 ± 0.27 mg/dL. The mean venous blood pH was 7.40 ± 0.05, and bicarbonate (HCO3^−^) was 21.50 ± 2.58 mmol/L. The mean serum uric acid level was 3.66 ± 1.46 mg/dL. Based on initial ultrasonographic evaluation, urinary stones were detected in 124 children (68.51%), while 57 (31.49%) had no sonographic evidence of stone formation (Table 1).

**Table 1:** Baseline demographic, clinical, and laboratory characteristics of the study population

Variables	*n* = 181
Gender—n (%)	
Female	102 (56.35%)
Male	79 (43.65%)
Consanguinity—n (%)	
No	158 (87.29%)
Yes	23 (12.71%)
Family history of stones—n (%)	
No	84 (46.41%)
Yes	97 (53.59%)
Age at first nephrology evaluation (month)—median (IQR)	6.00 (1.00–24.00)
Body weight (kg)—mean ± SD	7.25 ± 2.62
BUN (mg/dL)—mean ± SD	8.67 ± 4.94
Creatinine (serum) (mg/dL)—median (IQR)	0.43 (0.18–0.79)
Na (mmol/L)—mean ± SD	136.86 ± 2.35
K (mmol/L)—mean ± SD	4.75 ± 0.51
Ca (mg/dL)—mean ± SD	10.33 ± 0.92
P (mg/dL)—mean ± SD	5.48 ± 0.70
Mg (mg/dL)—mean ± SD	2.16 ± 0.27
Blood pH—mean ± SD	7.40 ± 0.05
HCO3 (mmol/L)—mean ± SD	21.50 ± 2.58
Uric acid (serum) (mg/dL)—mean ± SD	3.66 ± 1.46
Hypercalciuria—n (%)	
No	152 (83.98%)
Yes	29 (16.02%)
Hypocitraturia—n (%)	
No	151 (83.43%)
Yes	30 (16.57%)
USG—n (%)	
Stone present	124 (68.51%)

Data are presented as mean ± standard deviation (SD), median (interquartile range, IQR), or number (percentage), as appropriate

BUN, blood urea nitrogen; Na, sodium; K, potassium; Ca, calcium; P, phosphorus; Mg, magnesium; HCO₃⁻, bicarbonate; IQR, interquartile range; SD, standard deviation; USG, ultrasonography

Baseline demographic, clinical, and biochemical characteristics of the study groups are summarized in Table 2. Median age did not differ significantly among Group 1, Group 2, and Group 3 [8.0 (1.0–24.0), 4.0 (1.0–19.0), and 7.0 (1.0–23.0) months, respectively; *p* = 0.064]. Body weight and sex distribution were also comparable across the three groups (both *p* > 0.3).

**Table 2:** Baseline demographic, clinical, and biochemical characteristics of the study groups

Variable	Group 1 (*n* = 57)	Group 2 (*n* = 29)	Group 3 (*n* = 95)	p-value
Age (months)	8.0 (1.0–24.0)	4.0 (1.0–19.0)	7.0 (1.0–23.0)	0.064
Weight (kg)	7.7 (1.9–15.4)	6.9 (2.5–10.9)	7.5 (2.2–15.0)	0.381
Male sex, n (%)	32 (56.1%)	17 (58.6%)	56 (58.9%)	0.942
Hypercalciuria, n (%)	0 (0.0%)	29 (100.0%)	0 (0.0%)	-
Hypocitraturia, n (%)	9 (15.8%)	0 (0.0%)	21 (22.1%)	0.019
Vitamin D supplementation, n (%)	18 (31.6%)	12 (41.4%)	29 (30.5%)	0.540
Metabolic acidosis*, n/N (%)	9/24 (37.5%)	4/16 (25.0%)	13/41 (31.7%)	0.707
Hypokalemia (K < 3.5 mmol/L), n (%)	0 (0.0%)	0 (0.0%)	0 (0.0%)	-
BUN (mg/dL)	9.0 (3.0–34.0)	6.0 (2.6–15.0)	8.5 (2.4–25.0)	0.145
Creatinine (mg/dL)	0.44 (0.18–0.58)	0.42 (0.28–0.56)	0.43 (0.28–0.79)	0.632
Na (mmol/L)	137 (132–142)	137 (131–141)	137 (130–146)	0.821
K (mmol/L)	4.6 (3.5–6.5)	4.7 (4.0–5.7)	4.7 (3.6–6.2)	0.628
Ca (mg/dL)	10.3 (8.3–11.4)	10.6 (8.8–19.4)	10.3 (8.0–11.7)	0.347
P (mg/dL)	5.58 (2.6–7.0)	5.60 (4.0–6.5)	5.50 (3.8–7.0)	0.470
Mg (mg/dL)	2.30 (1.5–3.3)	2.13 (1.5–2.7)	2.13 (1.7–2.5)	0.193
Uric acid (mg/dL)	3.8 (2.0–18.8)	3.0 (1.4–7.0)	3.5 (1.5–7.8)	0.063

Data are presented as median (interquartile range) or number (percentage), unless otherwise indicated. *Metabolic acidosis was defined as venous bicarbonate* < *20 mmol/L and/or pH* < *7.35*

BUN, blood urea nitrogen; Ca, calcium; P, phosphorus; Mg, magnesium; Na, sodium; K, potassium; IQR, interquartile range

Hypercalciuria was present in all children in Group 2 and in none of the participants in Groups 1 and 3. Hypocitraturia was significantly more frequent in Group 1 (15.8%) and Group 3 (22.1%) compared with Group 2 (0%; *p* = 0.019). Rates of vitamin D supplementation did not differ significantly between the groups (*p* = 0.540).

Serum levels of BUN, creatinine, sodium, potassium, calcium, phosphorus, magnesium, and uric acid were comparable among the three groups (all *p* > 0.05). The prevalence of metabolic acidosis was similar across groups (*p* = 0.707), and hypokalemia was not observed in any of the study groups.

## Urinary Ca/Cit ratio in normocalciuric stone-free children

**Fig. 1:**
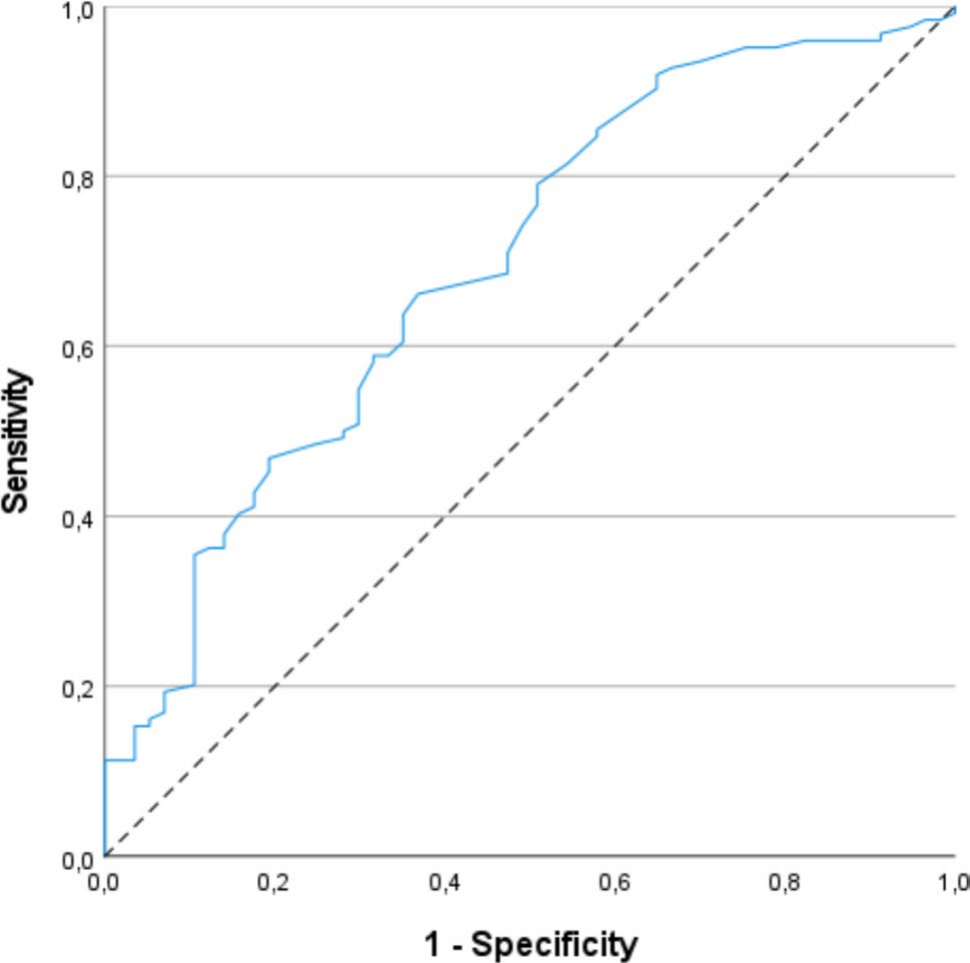
Receiver operating characteristic (ROC) curve of urinary calcium-to-citrate (Ca/Cit) ratio for predicting the presence of kidney stones. ROC, receiver operating characteristic; AUC, area under the curve; CI, confidence interval; Ca/Cit, urinary calcium-to-citrate ratio

Among normocalciuric stone-free controls (Group 1), the median spot Ca/Cit ratio was 0.17 (IQR 0.08–0.35) mg/mg, corresponding approximately to 0.81 (0.38–1.68) mmol/mmol. This distribution was used as the reference range for Ca/Cit in children aged 1–24 months, and subsequent analyses of stone risk were interpreted in relation to this range (Fig. 1).

## Age distribution and effect of age on urinary Ca/Cit ratio

**Fig. 2:**
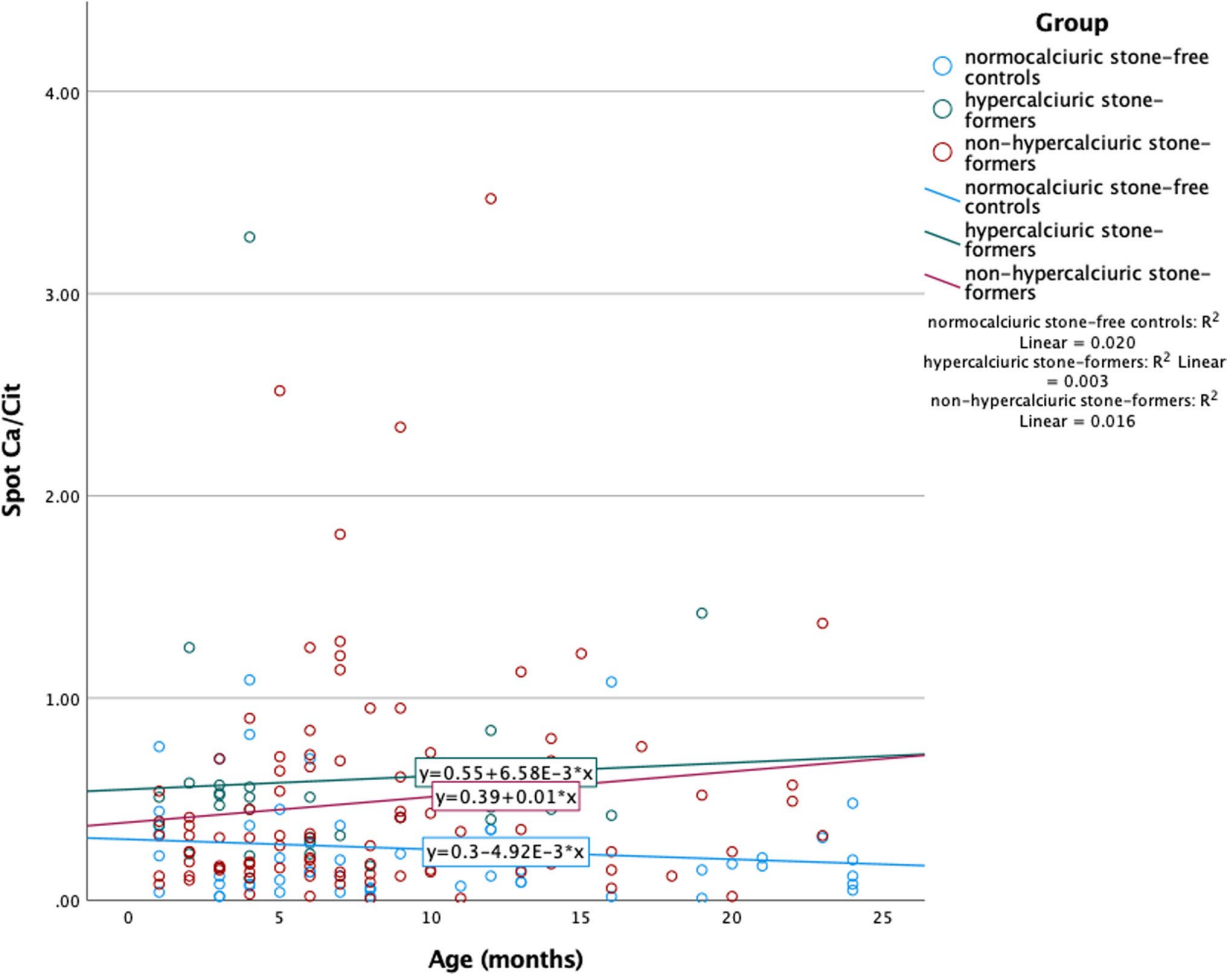
Scatter plot of urinary calcium-to-citrate (Ca/Cit) ratio versus age by study group in children aged 1–24 months. Ca/Cit, urinary calcium-to-citrate ratio

Age distributions were similar across the three groups (Table 2). In a Spearman correlation analysis including all 181 children, age was not significantly correlated with the Ca/Cit ratio (ρ = 0.002, *p* = 0.980). Consistent with this finding, visual inspection of the scatter plot of Ca/Cit versus age (Fig. 2) did not demonstrate any discernible age-related trend. These results suggest that the observed differences in Ca/Cit ratios between the study groups were not driven by age within the 1–24-month range. When Spearman correlation analyses were performed separately within each study group, no significant association was observed between age and the Ca/Cit ratio in any group. The correlation coefficients were weak and non-significant for Group 1 (ρ = –0.141, *p* = 0.297), Group 2 (ρ = 0.054, *p* = 0.786), and Group 3 (ρ = 0.125, *p* = 0.227).

## Comparison of urinary marker levels between study groups

The spot urinary Ca/Cr was significantly higher in Group 2 compared to Group 1 (1.03 vs. 0.19, *p* < 0.001) and Group 3 (1.03 vs. 0.29, *p* < 0.001); both comparisons remained statistically significant after Bonferroni correction. Although Group 3 had higher values than Group 1 (0.29 vs. 0.19, *p* = 0.028), this difference did not reach statistical significance after adjustment. The spot Cit/Cr was also elevated in Group 2 compared to Group 1 (2596.0 vs. 981.4, *p* < 0.001) and Group 3 (2596.0 vs. 981.0, *p* < 0.001); both comparisons remained statistically significant after correction. There was no significant difference between Group 1 and Group 3. For the Ca/Cit ratio, Group 2 had significantly higher values than Group 1 (0.46 vs. 0.17, *p* < 0.001), which remained significant after correction. Group 1 also had lower values than Group 3 (0.17 vs. 0.31, *p* = 0.013), but this did not reach the adjusted threshold for statistical significance. The spot UA/Cr was slightly higher in Group 2 compared to Group 1 (1.56 vs. 1.28, *p* = 0.041); however, this difference did not remain significant after correction. No significant differences were observed in Ox/Cr across the groups (*p* = 0.523). All pairwise comparisons were analyzed using the Mann–Whitney U test, with Bonferroni correction applied for multiple comparisons (adjusted significance threshold: *p* < 0.0033) (see Tables 3 and 4).

**Table 3:** Comparison of urinary marker levels between study groups

Parameter	Group 1 (*n* = 57)	Group 2 (*n* = 29)	Group 3 (*n* = 95)	*p*-value
Spot Ca/Cr	0.19 (0.09–0.33)	1.03 (0.83–1.31)	0.29 (0.17–0.46)	< 0.001
Spot Ox/Cr	69.15 (54.12–151.72)	82.15 (49.18–178.51)	71.01 (43.40–129.72)	0.523
Spot Cit/Cr	981.4 (554.4–1475.9)	2596.0 (1504.8–3470.0)	981.0 (437.5–1729.3)	< 0.001
Spot UA/Cr	1.28 (1.08–1.57)	1.56 (1.36–1.71)	1.38 (1.04–1.62)	0.041
Spot Ca/Cit	0.17 (0.08–0.35)	0.46 (0.31–0.56)	0.31 (0.15–0.65)	< 0.001

Data are presented as median (interquartile range). Spot Ox/Cr and Spot Cit/Cr are expressed as milligrams of solute per gram of creatinine (mg/g creatinine), whereas Spot Ca/Cr and Spot UA/Cr are expressed as milligrams of solute per milligram of creatinine (mg/mg creatinine). The Ca/Cit ratio in spot urine was calculated by dividing the concentration of calcium (mg/dL) by the concentration of citrate (mg/dL)

Ca, calcium; Cit, citrate; Ox, oxalate; UA, uric acid; Cr, creatinine; Ca/Cr, urinary calcium-to-creatinine ratio; Ox/Cr, urinary oxalate-to-creatinine ratio; Cit/Cr, urinary citrate-to-creatinine ratio; UA/Cr, urinary uric acid-to-creatinine ratio; Ca/Cit, urinary calcium-to-citrate ratio; IQR, interquartile range

**Table 4:** Post-hoc Mann–Whitney U test summary of pairwise comparisons between groups

Parameter	Median [IQR]	p	Adjusted p-value
Spot Ca/Cr			
Group 1 vs. Group 2	0.19 (0.01–0.57) vs. 1.03 (0.56–3.04)	< 0.001	< 0.001
Group 1 vs. Group 3	0.19 (0.01–0.57) vs. 0.29 (0.01–0.77)	0.008	< 0.001
Group 2 vs. Group 3	1.03 (0.56–3.04) vs. 0.29 (0.01–0.77)	< 0.001	< 0.001
Spot Cit/Cr			
Group 1 vs. Group 2	981.37 (11.95–4167.97) vs. 2596.0 (456.62–8617.65)	< 0.001	< 0.001
Group 1 vs. Group 3	981.37 (11.95–4167.97) vs. 981.0 (58.42–5875)	0.967	1.000
Group 2 vs. Group 3	2596.0 (456.62–8617.65) vs. 981.0 (58.42–5875)	< 0.001	< 0.001
Spot UA/Cr			
Group 1 vs. Group 2	1.28 (0.67–11.32) vs. 1.56 (0.58–2.33)	0.013	0.039
Group 1 vs. Group 3	1.28 (0.67–11.32) vs. 1.38 (0.50–4.77)	0.457	1.000
Group 2 vs. Group 3	1.56 (0.58–2.33) vs. 1.38 (0.50–4.77)	0.038	0.114
Spot Ca/Cit			
Group 1 vs. Group 2	0.17 (0.01–1.09) vs. 0.46 (0.11–3.28)	< 0.001	< 0.001 0.002
Group 1 vs. Group 3	0.17 (0.01–1.09) vs. 0.31 (0.01–3.47)	< 0.001	
Group 2 vs. Group 3	0.46 (0.11–3.28) vs. 0.31 (0.01–3.47)	0.038	0.114

Kruskal Wallis Test. A Bonferroni correction was applied to adjust for multiple comparisons. Ca, calcium; Cit, citrate; Ox, oxalate; UA, uric acid; Cr, creatinine; IQR, interquartile range

## Diagnostic performance of the spot urinary Ca/Cit ratio

The distribution of high versus low Ca/Cit values according to stone status is shown in Table 5 using a Ca/Cit cut-off of 0.23 mg/mg. A Ca/Cit ratio ≥ 0.23 mg/mg was observed in 82 of 124 children with kidney stones (66.1%) and in 21 of 57 children without stones (36.8%). Conversely, a Ca/Cit ratio < 0.23 mg/mg was present in 42 stone-forming children (33.9%) and in 36 non–stone-forming children (63.2%) (Table 5). The diagnostic accuracy of the spot Ca/Cit ratio for predicting the presence of urinary stones was assessed using ROC analysis (Table 6, Fig. 1). The AUC was 0.695 (95% CI: 0.613–0.785; *p* < 0.001), indicating moderate discriminative power. At a cut-off value of > 0.23 mg/mg (≈ 1.10 mmol/mmol), sensitivity and specificity were 66.1% and 63.2%, respectively, with a positive predictive value (PPV) of 77.2% and a negative predictive value (NPV) of 51.9%. Lower cut-off thresholds improved sensitivity at the expense of specificity: at > 0.155 mg/mg, sensitivity was 79.0% and specificity was 49.1%, while at > 0.125 mg/mg, sensitivity increased to 85.5% with a specificity of 42.1%.

**Table 5:** Distribution of urinary Ca/Cit ratio according to the presence of kidney stones in children under 2 years of age

Ca/Cit (mg/mg)	No stones (*n* = 57)		Stones present (*n* = 124)		Total (*n* = 181)		*p*
	n	%	n	%	n	%	
< 0.23 mg/mg	36	(63.16)	42	(33.87)	78	(43.09)	< 0.001
≥ 0.23 mg/mg	21	(36.84)	82	(66.13)	103	(56.91)	

*Chi-Square Test*

Ca/Cit, urinary calcium-to-citrate ratio

**Table 6:** Diagnostic accuracy of the spot urinary Ca/Cit ratio for predicting urinary stones

Parameter	Area	Std. Error	Asymptotic Sig	Asymptotic 95% CI		Cut-off	Sensitivity	Specificity	PPV	NPV
				Lower Bound	Upper Bound					
Spot Ca/Cit	0.695	0.042	< 0.001	0.613	0.785	> 0.230 > 0.155 > 0.125	66.1% 79.0% 85.5%	63.2% 49.1% 42.1%	77.2% 76.3%	51.9% 57.1%

*Diagnostic performance was assessed using receiver operating characteristic (ROC) curve analysis. Additional cut-off values are shown to illustrate the trade-off between sensitivity and specificity at lower thresholds*

Ca/Cit, urinary calcium-to-citrate ratio; AUC, area under the curve; CI, confidence interval; PPV, positive predictive value; NPV, negative predictive value

## Discussion

This single-center retrospective cohort study involved 181 children aged 1–24 months who were evaluated for suspected urinary stone disease. The study found that the urinary Ca/Cit ratio exhibited moderate capability in differentiating between stone-forming and stone-free patients. A cut-off value of > 0.23 mg/mg (≈ 1.10 mmol/mmol) for urinary Ca/Cit was found to yield an AUC of 0.695 (95% CI 0.613–0.785), with 66.1% sensitivity, 63.2% specificity, and a positive predictive value of 77.2% for the presence of kidney stones. Utilizing normocalciuric, stone-free children (Group 1) as the reference population, the distribution of "normal" Ca/Cit values in this age group was [median 0.17 (IQR 0.08–0.35) mg/mg; ≈ 0.81 (0.38–1.68) mmol/mmol] and it was observed that Ca/Cit ratios were higher in both hypercalciuric stone-formers (Group 2) and non-hypercalciuric stone-formers (Group 3). Although spot Ca/Cr and Cit/Cr ratios also differed, particularly among hypercalciuric stone-formers, the Ca/Cit ratio more directly reflected the balance between urinary calcium load and citrate's inhibitory capacity.

Citrate is a known inhibitor of calcium stone formation, as it forms a complex with calcium and reduces free ionic calcium in the urine. In our cohort, children with higher Ca/Cit ratios had a higher prevalence of stones, which is consistent with the protective role of urinary citrate described in previous studies [4, 9]. Since the urinary calcium-to-citrate ratio does not include creatinine, it is unaffected by gender, age, or muscle mass, making it a potential parameter for stone risk assessment [6, 10]. Two separate studies conducted in Poland found significantly higher urinary calcium-to-citrate ratios in kidney stone patients compared to controls [10, 11]. In a cohort of 78 hypercalciuric non-stone-formers, 34 hypercalciuric stone-formers, and 149 healthy controls aged 5–18 years, Srivastava et al. demonstrated that random urinary calcium and calcium-to-citrate ratios effectively differentiated healthy children from those with stones [12]. Kompani et al. reported that the urinary calcium-to-citrate ratio effectively discriminated between stone-forming children and healthy controls aged 2–12 years and was higher in those with a family history of stones [6]. A Canadian study of children aged 8–10 years reported urinary calcium-to-citrate ratios of 0.68 mmol/mmol in non-stone-formers and 1.30 mmol/mmol in stone-formers [13]. Turudic et al. found that 24-h urinary calcium-to-citrate ratios in Croatian children aged 7–13 years were 3.42 mol/mmol in stone-formers and 1.06 mol/mmol in healthy controls [14]. To date, no literature specifically addresses urinary calcium-to-citrate ratios in children under 2 years. Our findings suggest that a urinary calcium-to-citrate ratio greater than 0.23 mg/mg (≈ 1.10 mmol/mmol) may serve as a useful adjunctive marker for identifying stone-forming children in this young age group.

## Limitations of the study

This study has several limitations that should be considered when interpreting the findings. First, its retrospective, single-center design and the fact that all participants were recruited from a tertiary pediatric nephrology referral clinic introduce potential selection bias and may limit the generalizability of our results to broader pediatric populations. Second, because 24-h urine collection is often impractical in infants and toddlers, we relied on spot urine samples to assess calcium and citrate excretion; although this approach reflects real-world clinical practice, spot measurements may not fully capture diurnal variation or day-to-day metabolic fluctuations. Third, the Ca/Cit cut-off value of > 0.23 mg/mg (≈ 1.10 mmol/mmol) was derived from a single-center cohort without external validation, and the relatively small size of the hypercalciuric stone-former group (Group 2) may have contributed to wider confidence intervals around some estimates. Consistent with this, the Ca/Cit ratio demonstrated only moderate discriminatory performance, with a fair AUC and a modest negative predictive value and should therefore be regarded as an adjunctive marker rather than a stand-alone diagnostic tool. Finally, potentially relevant clinical factors such as detailed dietary patterns, fluid intake, concomitant medications, and intercurrent infections were not systematically captured and could not be fully accounted for in our analyses. Prospective, multicenter studies with standardized metabolic evaluation and external validation of Ca/Cit thresholds are needed to confirm and refine these findings.

## Conclusions

In this single-center cohort of 181 children under two years of age evaluated for suspected urinary stone disease, it was found that the Ca/Cit ratio provides moderate discriminatory ability for the presence of kidney stones. A urinary Ca/Cit cut-off value of > 0.23 mg/mg (≈ 1.10 mmol/mmol) was identified as a means of identifying children at increased risk, and normocalciuric, stone-free infants and toddlers allowed for the characterization of an age-specific reference distribution for "normal" Ca/Cit values in this early period of life. The findings of the present study also suggest that an imbalance between urinary calcium and citrate—rather than hypercalciuria or hypocitraturia alone—may represent an important determinant of stone formation in early childhood.

When interpreted in the context of previous studies in older children, which have consistently reported higher Ca/Cit ratios in stone-formers than in controls, our data extend the potential utility of the Ca/Cit ratio to children under 2 years of age. Taken together, these observations lend support to the use of the Ca/Cit ratio as a valuable adjunctive marker within the metabolic evaluation of infants and toddlers with suspected stone disease, rather than as a stand-alone diagnostic test. The conduction of prospective multicenter studies with larger sample sizes is indicated to validate age-specific reference intervals and refine clinically useful cut-off values in this vulnerable age group.

## Supplementary Information

Below is the link to the electronic supplementary material.

Graphical abstract (PPTX 156 KB)

## Data Availability

Data are available upon reasonable request, subject to ethical approval.
