# Supplementary material for: Urinary calcium-to-citrate ratio predicts kidney stone risk in children under the age of two years
Source: Pediatr Nephrol. 2026 Feb 11;41(8):2499–507. doi: 10.1007/s00467-026-07191-z (PMC13337681; doi:10.1007/s00467-026-07191-z)
Supplement: Supplementary file 1 — Graphical abstract (PPTX 156 KB) [file 467_2026_7191_MOESM1_ESM.pptx]

## Slide 1
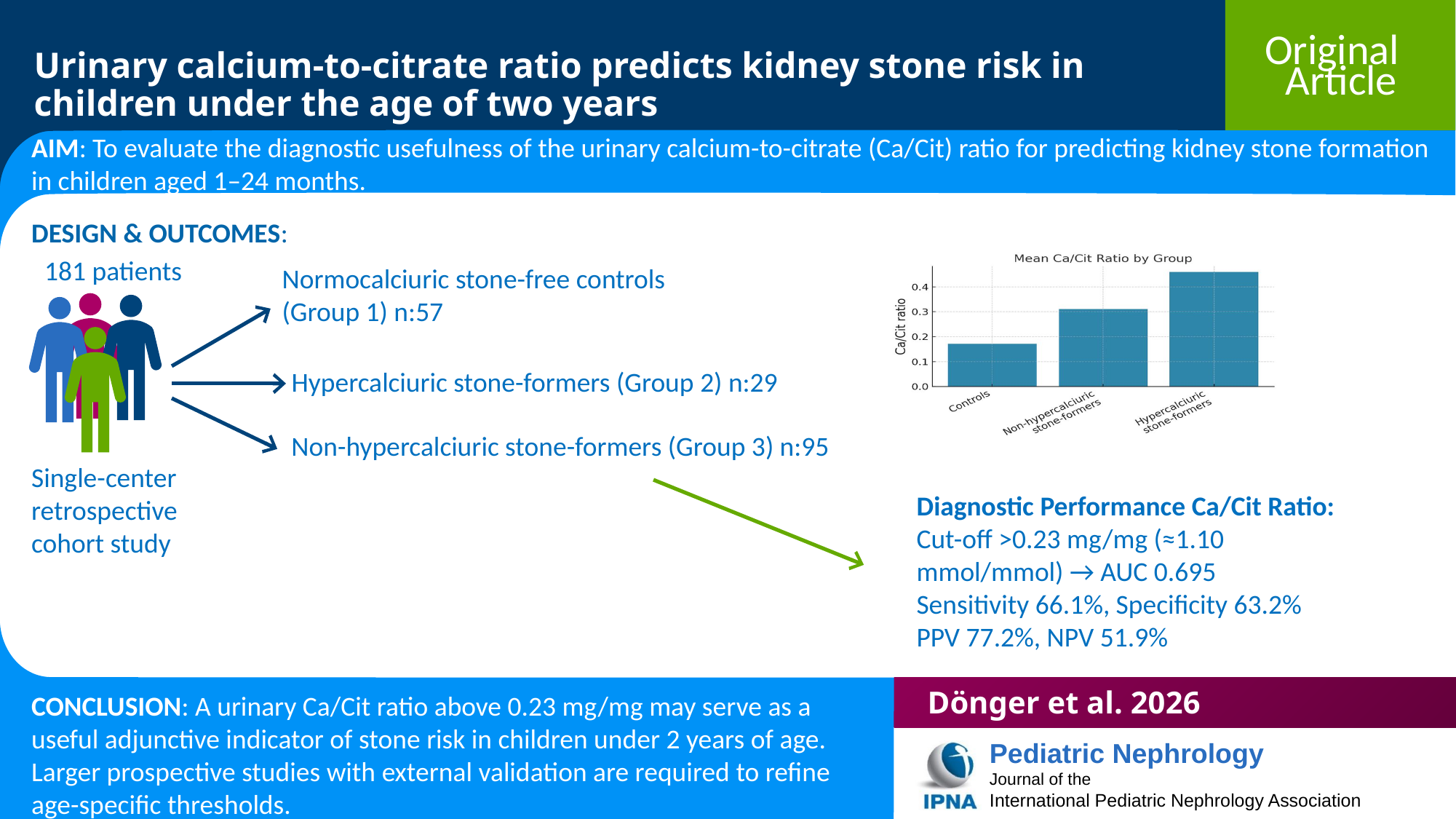

Urinary calcium-to-citrate ratio predicts kidney stone risk in children under the age of two years
AIM: To evaluate the diagnostic usefulness of the urinary calcium-to-citrate (Ca/Cit) ratio for predicting kidney stone formation in children aged 1–24 months.
DESIGN & OUTCOMES:
181 patients
Normocalciuric stone-free controls (Group 1) n:57
Hypercalciuric stone-formers (Group 2) n:29
Non-hypercalciuric stone-formers (Group 3) n:95
Single-center retrospective cohort study
Diagnostic Performance Ca/Cit Ratio:
Cut-off >0.23 mg/mg (≈1.10 mmol/mmol) → AUC 0.695
Sensitivity 66.1%, Specificity 63.2%
PPV 77.2%, NPV 51.9%
Dönger et al. 2026
CONCLUSION: A urinary Ca/Cit ratio above 0.23 mg/mg may serve as a useful adjunctive indicator of stone risk in children under 2 years of age. Larger prospective studies with external validation are required to refine age-specific thresholds.
